# A Rare Case of Hereditary Angioedema in a 7‐Year‐Old Girl: Presentation, Diagnosis, and Bridging the Management Gap

**DOI:** 10.1155/carm/9980409

**Published:** 2026-07-31

**Authors:** Amir Hasan Farzaneh, Abbas Dabbaghzadeh

**Affiliations:** ^1^ Department of Clinical Pharmacy, School of Pharmacy, Mazandaran University of Medical Sciences, Sari, Iran, mazums.ac.ir; ^2^ Pediatric Infectious Diseases Research Center, Communicable Diseases Institute, Mazandaran University of Medical Sciences, Sari, Iran, mazums.ac.ir

**Keywords:** bradykinin, C1 inhibitor, case report, fresh frozen plasma, hereditary angioedema, pediatrics

## Abstract

Hereditary angioedema (HAE) is a rare, potentially life‐threatening genetic disorder characterized by recurrent episodes of localized subcutaneous or submucosal swelling resulting from C1 esterase inhibitor deficiency or dysfunction. We report the case of a 7‐year‐old girl who presented with acute left‐sided facial and upper lip edema that initially appeared as periorbital discoloration resembling ecchymosis following an upper respiratory tract infection. Her history included recurrent self‐limited swelling episodes and an initially unrecognized family history of angioedema. Physical examination demonstrated nonpitting, nonpruritic facial edema without signs of airway compromise. Routine laboratory investigations were unremarkable. Complement testing subsequently confirmed HAE Type I, demonstrating markedly reduced C1 esterase inhibitor antigen and C4 levels with normal C1q concentrations. Because specific C1‐INH replacement therapy was unavailable, the patient received fresh frozen plasma (approximately 15 mL/kg), resulting in complete clinical resolution within 3 days. This case highlights the diagnostic challenges of pediatric HAE, particularly when clinical manifestations mimic more common allergic, infectious, or traumatic conditions. It also illustrates the importance of early recognition, timely complement testing, and family screening for establishing the diagnosis. Furthermore, this report demonstrates that fresh frozen plasma may serve as an effective therapeutic alternative for acute attacks in resource‐limited settings where targeted therapies are inaccessible. Early diagnosis, patient education, trigger avoidance, and appropriate long‐term follow‐up remain essential to reduce morbidity and prevent potentially life‐threatening complications.

## 1. Introduction

Hereditary angioedema (HAE) is a rare, life‐threatening, autosomal dominant disorder most commonly caused by a deficiency (Type I) or dysfunction (Type II) of C1 esterase inhibitor (C1‐INH). This defect leads to dysregulated bradykinin production, resulting in recurrent episodes of localized, nonpruritic subcutaneous or submucosal swelling. While the overall prevalence is estimated at 1 in 50,000, pediatric cases present unique diagnostic and therapeutic challenges [1, 2]. Symptoms often manifest during childhood or adolescence, yet diagnosis is frequently delayed for years, with reports of a median diagnostic lag exceeding a decade in adult cohorts [1, 3]. This delay stems from several factors: Presentations in children are often atypical, with isolated abdominal pain or peripheral edema mimicking common conditions like gastroenteritis or allergic reactions [4]. Furthermore, the absence of urticaria and pruritus distinguishes HAE from the more prevalent mast cell–mediated angioedema. Compounding this, up to 25% of cases result from de novo *SERPING1* gene mutations, meaning a suggestive family history is absent in a significant minority of patients [1, 5]. These diagnostic hurdles can lead to unnecessary interventions, heightened patient and family anxiety, and a significant impairment in quality of life before appropriate management is initiated [6]. Timely diagnosis, confirmed by complement testing (C4, C1‐INH antigen and function), is therefore critical to prevent life‐threatening laryngeal edema and guide effective management [1]. Treatment paradigms are evolving, with targeted on‐demand and prophylactic therapies, yet access to these remains limited in many resource‐constrained settings [7]. This report describes a confirmed case of HAE Type I in a 7‐year‐old girl. It aims to highlight the clinical clues to early diagnosis, underscore the pivotal role of confirmatory laboratory evaluation, and discuss the practicalities of acute and long‐term management in an environment where first‐line targeted therapies are not readily available.

## 2. Case Presentation

A 7‐year‐old girl presented with a 24‐h history of progressive left‐sided facial and upper lip swelling that initially appeared as periorbital discoloration resembling ecchymosis before evolving into nonpitting edema. During the week preceding admission, she experienced worsening rhinorrhea and sore throat but denied dyspnea, drooling, abdominal pain, fever, or gastrointestinal symptoms. One week prior, she had developed self‐limited swelling of the hand during an upper respiratory tract infection.

Her past medical history was notable for normal growth and development following cesarean delivery at 36 weeks of gestation, complete immunization, and hospitalization at 3 years of age for fever and abdominal swelling that had been attributed to a viral illness. Two months before the current admission, a separate episode of transient hand swelling prompted clinical suspicion of HAE. However, because the symptoms resolved spontaneously and access to specialized immunologic evaluation was limited, confirmatory complement testing was not obtained at that time.

**FIGURE 1:**
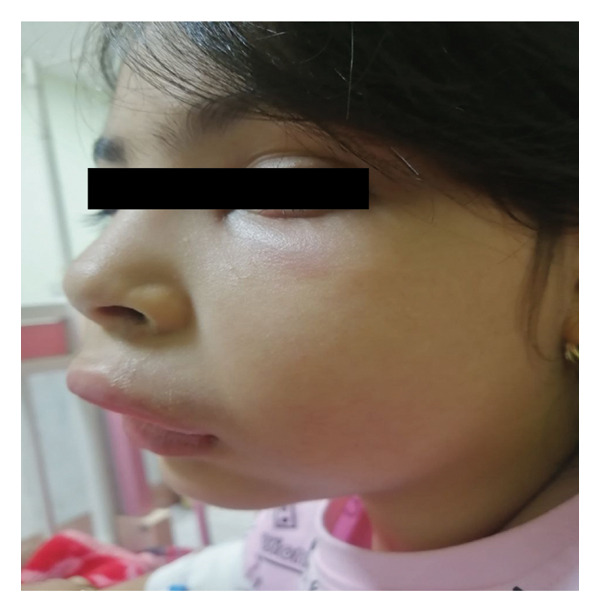
Left‐sided facial edema, nonpitting, nonpruritic, with mild periorbital erythema.

**FIGURE 2:**
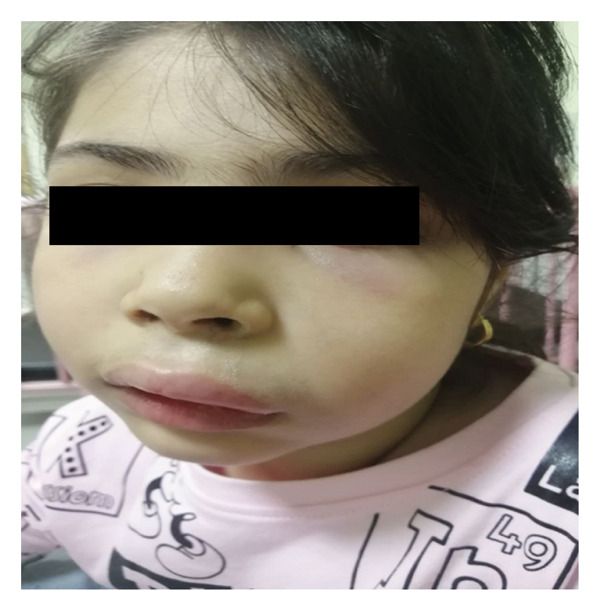
Left‐sided facial edema, nonpitting, nonpruritic, with mild periorbital erythema.

On examination, the patient appeared comfortable, alert, and nontoxic. Vital signs included a blood pressure of 100/55 mmHg, heart rate of 108 beats/min, respiratory rate of 29 breaths/min, oxygen saturation of 98% on room air, and body temperature of 36.2°C. Physical examination revealed unilateral left‐sided facial edema involving the periorbital region and upper lip. The swelling was nonpitting, nonpruritic, and associated with mild erythema (Figures 1 and 2).

Clear rhinorrhea was present. No urticaria, tongue swelling, stridor, wheezing, or signs of airway compromise were observed. Cardiovascular, abdominal, and neurological examinations were unremarkable. Routine laboratory investigations were largely unremarkable (Table 1). Given the recurrent nature of symptoms and the subsequently recognized maternal history of angioedema, a comprehensive complement evaluation was performed following stabilization. The results demonstrated markedly decreased C1 esterase inhibitor antigen levels and reduced C4 concentrations with normal C1q levels, consistent with HAE Type I (Table 2).

**TABLE 1:** Admission laboratory results.

Parameter	Value	Normal range
WBC (× 10^3^/μL)	16.1	4.5–13.5
Lymphocytes (%)	24	20–40
Neutrophils (%)	65	40–60
Hemoglobin (g/dL)	11.3	11.5–14.5
RBC (× 10^6^/μL)	4.8	4.0–5.0
MCV (fL)	78	75–87
MCH (pg)	23.5	24–30
Platelets (× 10^3^/μL)	333	150–400
Urea (mg/dL)	14	10–40
Creatinine (mg/dL)	0.4	0.3–0.7
Sodium (mEq/L)	139	135–145
Potassium (mEq/L)	4.0	3.5–5.0

*Note:* The patient was admitted for close observation because of the risk of progression to airway involvement. Management consisted of fasting status, intravenous hydration, and administration of fresh frozen plasma (approximately 15 mL/kg; total dose 300 mL) because specific C1‐INH concentrate was not available. A pediatric intensive care consultation was obtained, and emergency airway management resources were kept readily available.

**TABLE 2:** Confirmatory complement and C1 inhibitor profile.

Test	Result	Normal range	Interpretation
C1 esterase inhibitor antigen	0.10 g/L^∗^	0.22–0.38 g/L	Low
Complement C4	7.93 mg/dL^∗^	10–40 mg/dL	Low
Complement C3	128.20 mg/dL	75–180 mg/dL	Normal
C1 esterase inhibitor functional assay	51%	> 67% normal	Equivocal/borderline low
Complement total (CH50)	180.0%^∗^	51%–150%	High
Complement C1q	153.20 mg/L	118–244 mg/L	Normal

^∗^Confirmed by repeated analysis.

**FIGURE 3:**
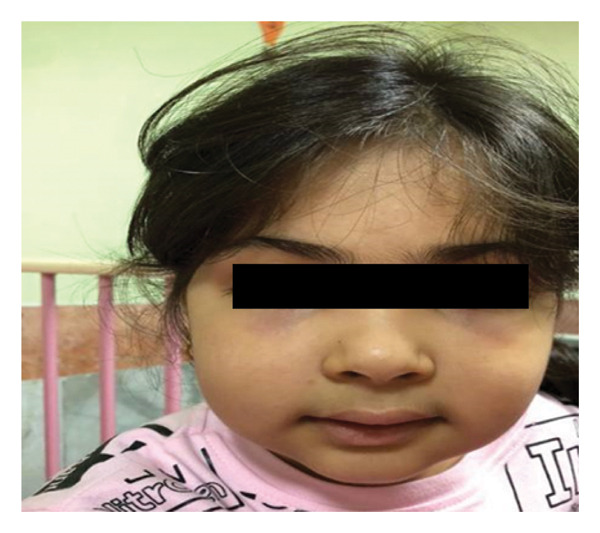
Resolution of facial edema on Day 3.

Substantial clinical improvement was observed within 24 h. Facial swelling progressively regressed, oral intake was resumed, and no respiratory complications occurred. By hospital day 3, the edema had resolved completely (Figure 3). The patient was discharged with a confirmed diagnosis of HAE Type I. Education regarding disease recognition, trigger avoidance, emergency management, and specialist follow‐up was provided. Subsequent family screening revealed that the patient’s mother also had previously undiagnosed HAE Type I.

## 3. Discussion

This case is noteworthy for several reasons. First, HAE remains underrecognized in young children, particularly in regions where access to specialized diagnostic testing and targeted therapies is unavailable. Second, the patient’s initial presentation with periorbital discoloration and unilateral facial swelling could easily mimic trauma, cellulitis, insect bites, or allergic disease, potentially delaying recognition of an underlying bradykinin‐mediated disorder. Third, successful treatment was achieved using fresh frozen plasma (FFP) in the absence of disease‐specific therapies, highlighting a practical management strategy applicable in many resource‐limited settings. Finally, diagnosis of the index patient led directly to identification of previously unrecognized HAE in her mother, emphasizing the importance of family screening.

The presentation of facial swelling following an upper respiratory infection is consistent with established triggers of HAE attacks. Viral infections, emotional stress, trauma, and medical procedures are among the most commonly reported precipitating factors [8]. The absence of urticaria and pruritus, together with recurrent self‐limited episodes, strongly supported a bradykinin‐mediated rather than mast cell–mediated mechanism.

An unusual and clinically important aspect of this case was the initial appearance of periorbital discoloration resembling ecchymosis. Such manifestations may be mistaken for facial trauma, insect bites, infectious processes, or allergic reactions, potentially leading to misdiagnosis and unnecessary investigations. Recent pediatric guidance emphasizes early recognition and treatment initiation to reduce cumulative disease burden and improve quality of life in affected children [2]. Therefore, awareness of this atypical presentation may facilitate earlier recognition of HAE in pediatric patients.

### 3.1. Diagnostic Challenges and Unique Features

The diagnosis of HAE is often delayed in children due to symptom overlap with common conditions. Abdominal pain may be misinterpreted as gastroenteritis or appendicitis, leading to unnecessary surgeries [9]. Peripheral swelling is frequently mislabeled as an allergic reaction. A high index of suspicion is required, particularly for recurrent, nonpruritic angioedema not responding to antihistamines or steroids. As demonstrated here, laboratory confirmation is paramount [1]. The hallmark finding is a low C4 level during and between attacks [10]. Definitive diagnosis requires assessment of C1‐INH antigen and function. Our patient’s profile, low antigenic C1‐INH and low C4 with normal C1q, is diagnostic for HAE Type I [1, 10]. Genetic testing of the *SERPING1* gene, while not essential for diagnosis in a clear biochemical case, can be useful for family screening and identifying de novo mutations [11].

### 3.2. Acute and Long‐Term Management Strategies

The cornerstone of acute attack management, especially for laryngeal or severe abdominal involvement, is early intervention with targeted therapies [1]. First‐line agents include plasma‐derived or recombinant C1‐INH concentrate, the bradykinin B2 receptor antagonist icatibant, and the kallikrein inhibitor ecallantide. In settings where these are inaccessible, FFP serves as a critical alternative, as it contains C1‐INH. However, FFP use requires caution due to a theoretical risk of exacerbating attacks by providing additional substrate for bradykinin generation (Table 3). For this patient, FFP was effective and well tolerated [10]. It is important to note that while epinephrine was available on standby during this admission for potential airway compromise, it is not a disease‐specific treatment for bradykinin‐mediated angioedema, and its role is supportive and adjunctive at best. Similarly, while famotidine was prescribed at discharge in this case as a readily available agent, its use for HAE prophylaxis is not evidence‐based, and it should not be considered a substitute for approved prophylactic therapies when available.

**TABLE 3:** Comparison of acute therapies for HAE in children.

Therapy	Mechanism	Pediatric use	Limitations
C1‐INH concentrate	Replaces deficient C1‐INH	FDA‐approved for ≥ 2 years	Cost, availability
FFP	Supplies C1‐INH	Accessible, low cost	Risk of exacerbation
Icatibant	Bradykinin B2 antagonist	Limited pediatric data	Subcutaneous injection
Supportive care	Symptomatic relief	Always adjunctive	Does not treat underlying cause

Long‐term prophylaxis should be individualized based on attack frequency, severity, and impact on quality of life. Options include regular intravenous or subcutaneous C1‐INH infusions, the subcutaneous monoclonal antibody lanadelumab (which inhibits kallikrein), and the oral agent berotralstat [2, 10]. Attenuated androgens and antifibrinolytics like tranexamic acid are less favored in children due to side‐effect profiles but may be considered in resource‐limited contexts. In particular, tranexamic acid could be considered for long‐term prophylaxis in cases of frequent attacks when first‐line options are unavailable, despite its limited efficacy and potential side effects. Comprehensive care must also include patient and family education, an emergency action plan, psychological support, and trigger avoidance (e.g., estrogen‐containing medications and ACE inhibitors) [1, 2, 10].

### 3.3. The Importance of Family Screening and Genetic Counseling

Given the autosomal dominant inheritance, screening all first‐degree relatives of an index case is mandatory [1, 10]. This involves measuring C4, C1‐INH antigen, and C1‐INH function. As highlighted in the literature, family screening can lead to presymptomatic diagnosis, drastically reducing diagnostic delay and anxiety [11]. In this case, subsequent screening of the mother confirmed a diagnosis of HAE Type I, reinforcing the importance of this practice. Genetic counseling helps families understand inheritance patterns and reproductive options. Engagement with patient support organizations, such as HAE International (HAEi), can provide invaluable resources, education, and community support. In Iran, an active HAE patient association operates under the HAEi network, offering local support for affected families.

In summary, this report confirms HAE Type I in a young child through classic biochemical findings, emphasizing that diagnosis should be considered in any child with recurrent, nonpruritic angioedema. While diagnostic delays are common, a systematic approach involving complement testing can yield a timely diagnosis. Management, even in resource‐limited environments, can be successful with available therapies like FFP for acute attacks and a focus on comprehensive, patient‐centered long‐term care. Early diagnosis and family screening are essential to mitigate morbidity and mortality from this potentially life‐threatening condition.

Patient Perspective: The patient’s mother expressed relief upon receiving a clear diagnosis after months of uncertainty. She felt more empowered to recognize early signs of an attack and seek appropriate care promptly. The family appreciated the thorough explanation provided by the medical team regarding the nature of the condition, the importance of trigger avoidance, and the emergency action plan. They reported confidence in the management plan moving forward, despite the challenges of accessing specialized treatments in their setting, and emphasized the value of continued education and support for families dealing with rare disorders.

## 4. Limitations

This report is limited by its nature as a single case study, which precludes generalizability of the findings. The short‐term follow‐up period also limits insights into the long‐term clinical course, attack frequency, and the effectiveness of the implemented management strategy over time. Furthermore, while FFP was used successfully here, its variable composition and theoretical risks warrant caution, and its use should not overshadow the need to improve access to targeted first‐line therapies globally.

## Funding

No funding was received for this article.

## Consent

Written informed consent was obtained from the parents for publication of this case report and any accompanying images.

## Conflicts of Interest

The authors declare no conflicts of interest.

## Data Availability

The datasets for the current study are available from the corresponding author upon reasonable request.
